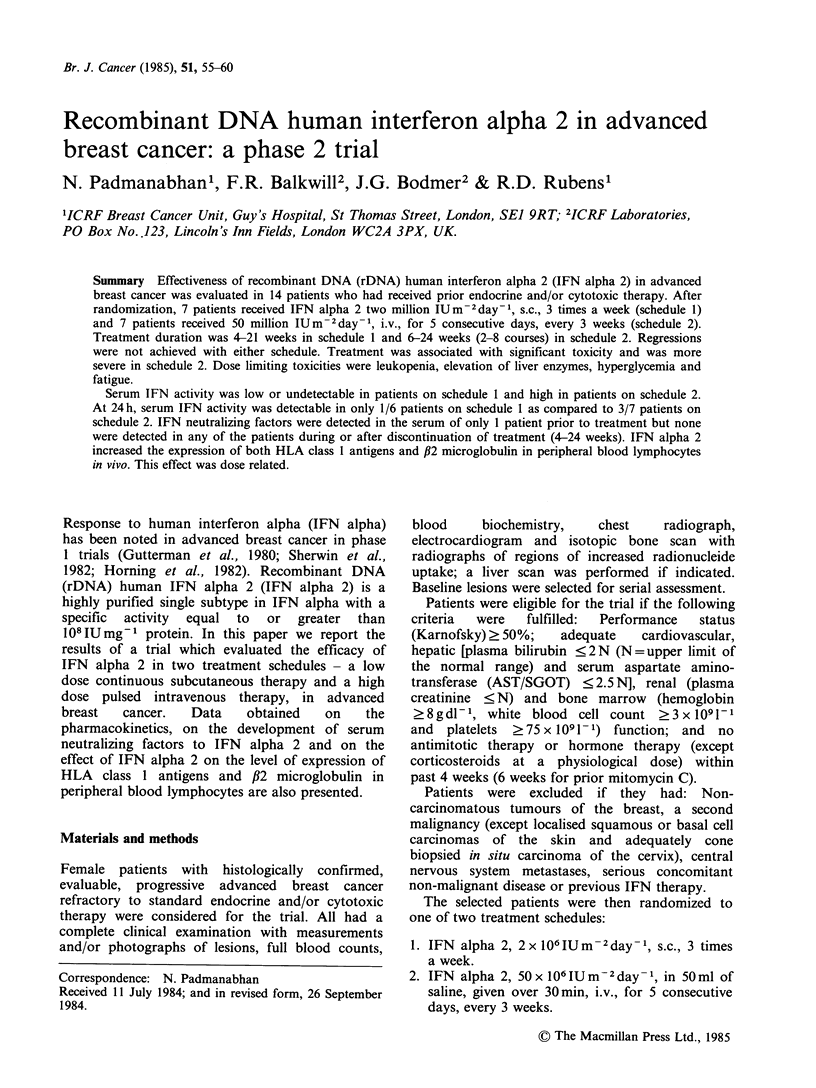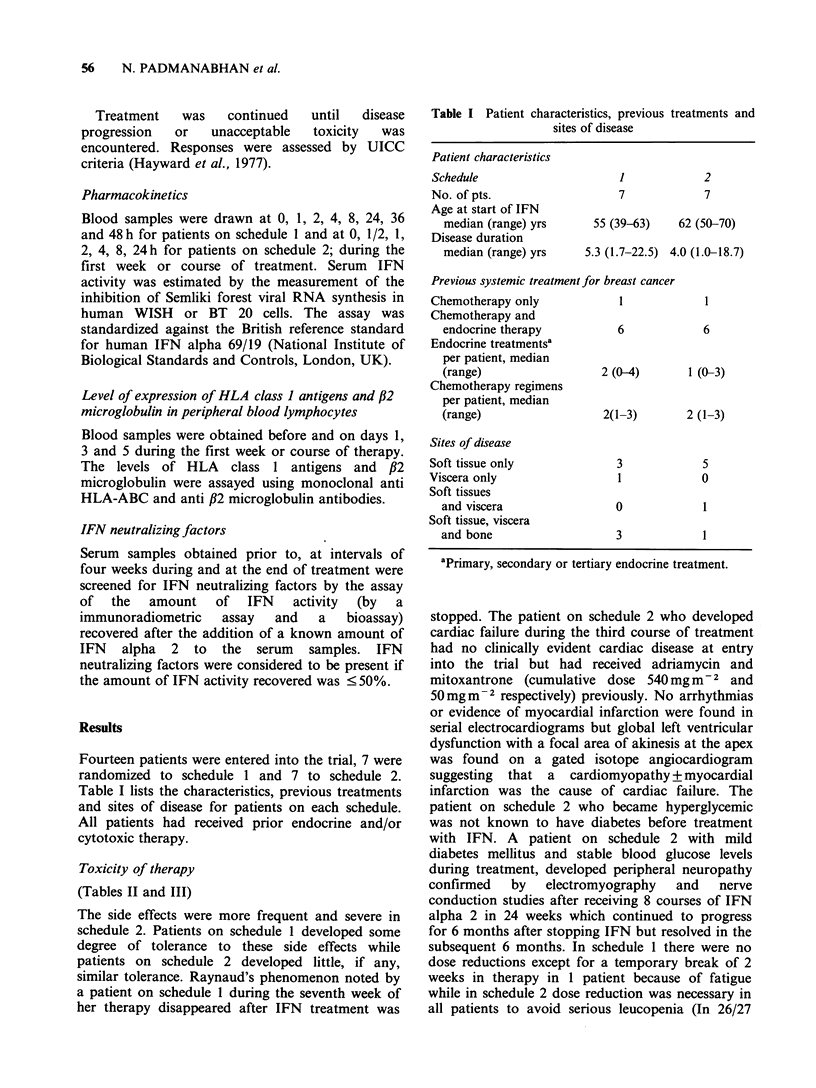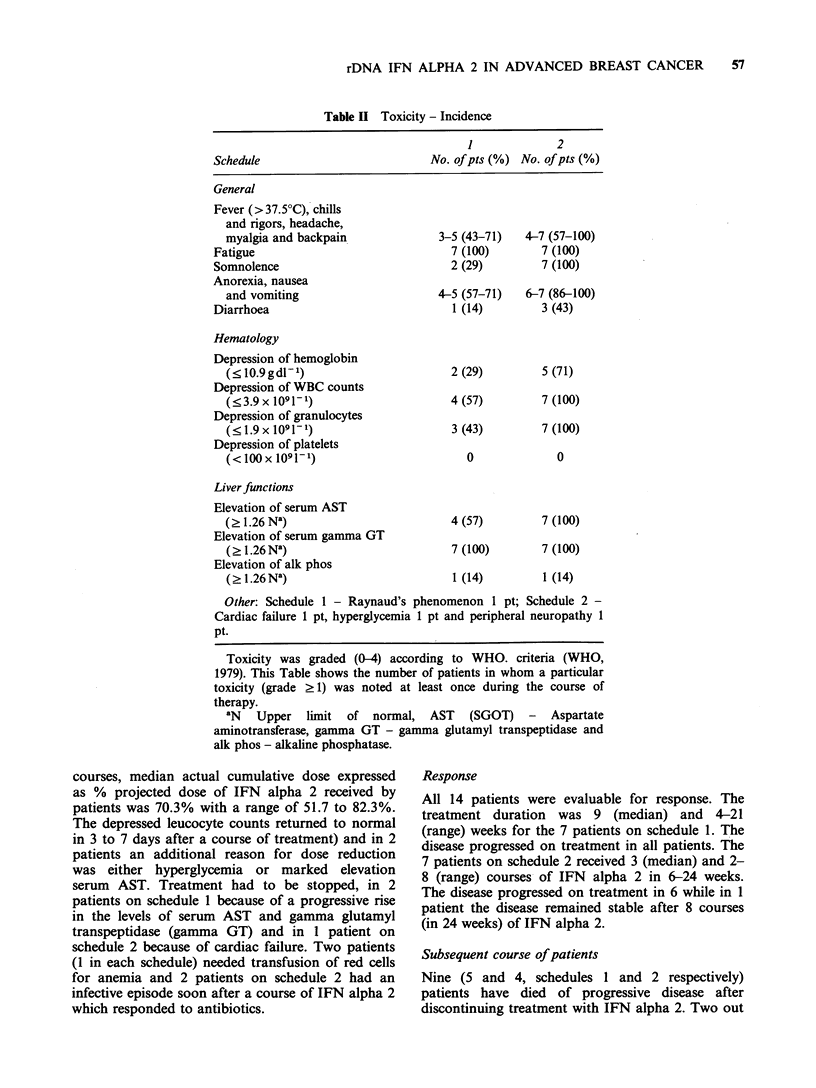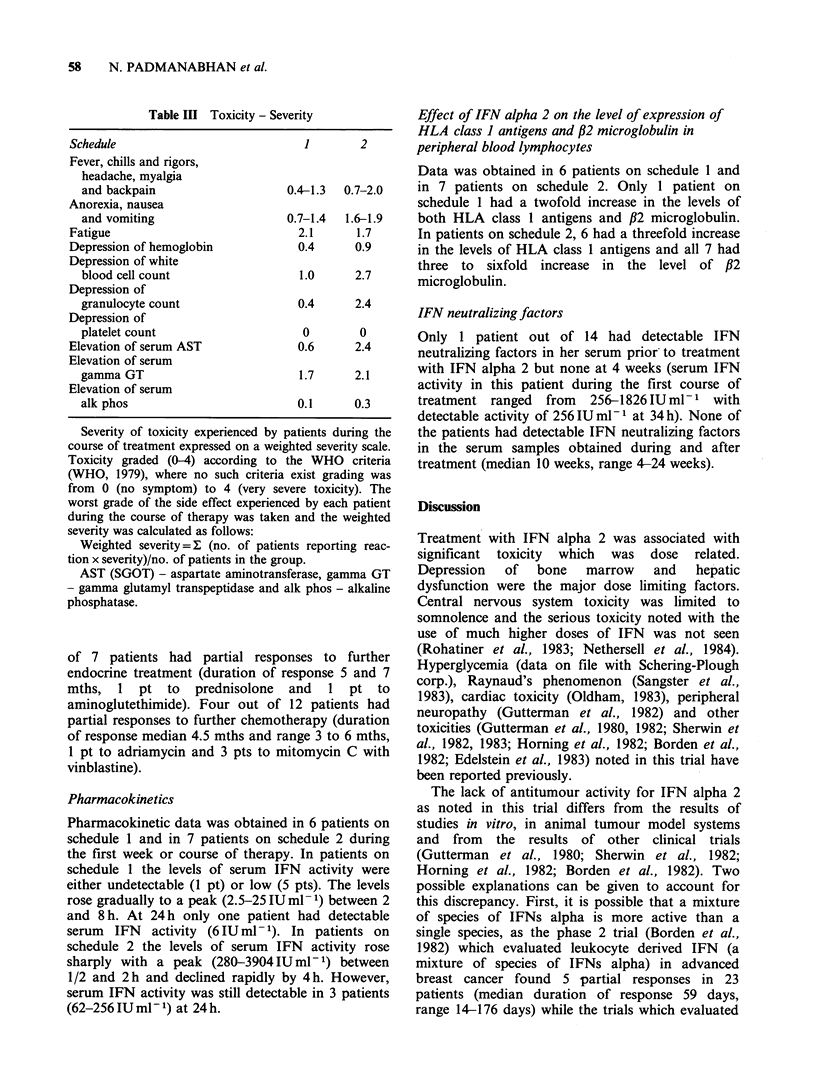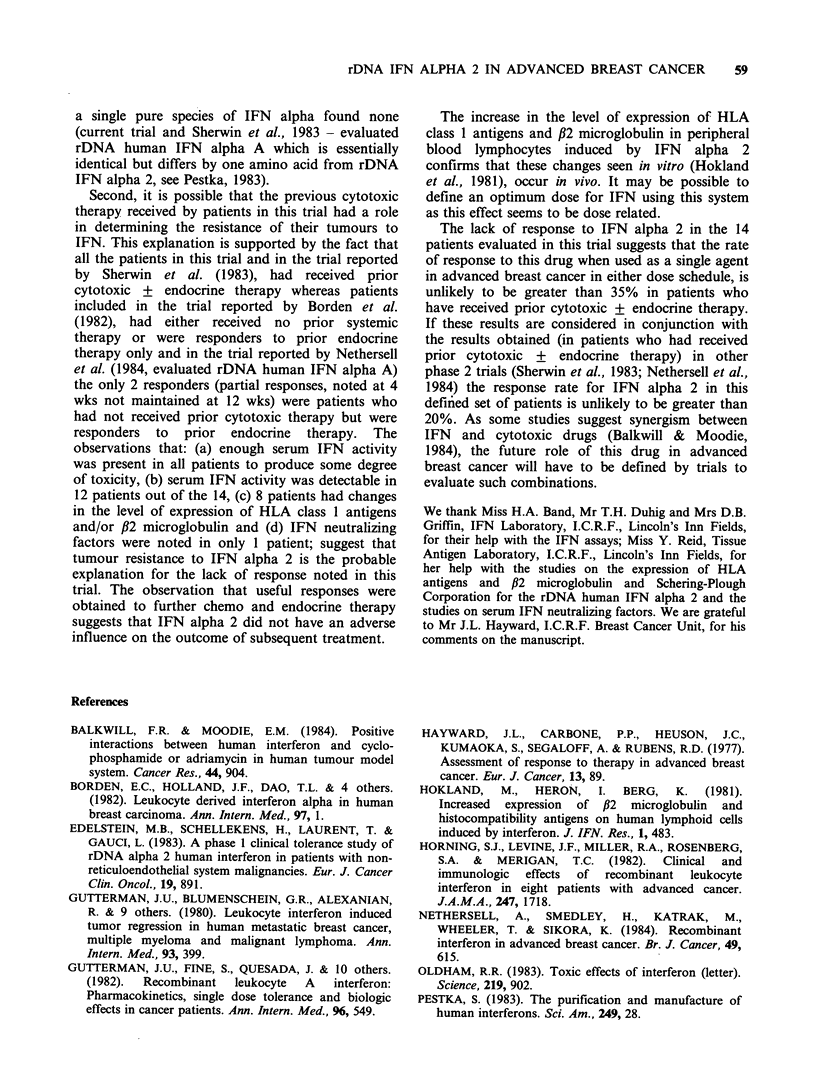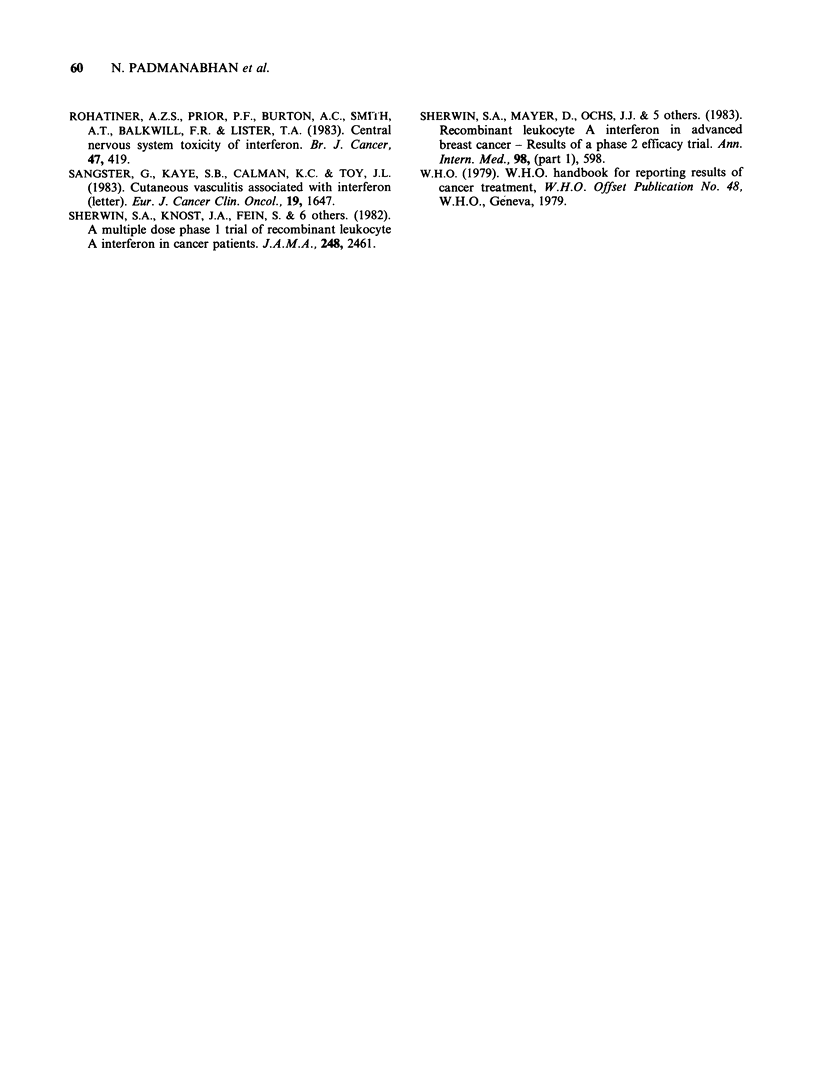# Recombinant DNA human interferon alpha 2 in advanced breast cancer: a phase 2 trial.

**DOI:** 10.1038/bjc.1985.8

**Published:** 1985-01

**Authors:** N. Padmanabhan, F. R. Balkwill, J. G. Bodmer, R. D. Rubens

## Abstract

Effectiveness of recombinant DNA (rDNA) human interferon alpha 2 (IFN alpha 2) in advanced breast cancer was evaluated in 14 patients who had received prior endocrine and/or cytotoxic therapy. After randomization, 7 patients received IFN alpha 2 two million IU m-2 day-1, s.c., 3 times a week (schedule 1) and 7 patients received 50 million IU m-2 day-1, i.v., for 5 consecutive days, every 3 weeks (schedule 2). Treatment duration was 4-21 weeks in schedule 1 and 6-24 weeks (2-8 courses) in schedule 2. Regressions were not achieved with either schedule. Treatment was associated with significant toxicity and was more severe in schedule 2. Dose limiting toxicities were leukopenia, elevation of liver enzymes, hyperglycemia and fatigue. Serum IFN activity was low or undetectable in patients on schedule 1 and high in patients on schedule 2. At 24 h, serum IFN activity was detectable in only 1/6 patients on schedule 1 as compared to 3/7 patients on schedule 2. IFN neutralizing factors were detected in the serum of only 1 patient prior to treatment but none were detected in any of the patients during or after discontinuation of treatment (4-24 weeks). IFN alpha 2 increased the expression of both HLA class 1 antigens and beta 2 microglobulin in peripheral blood lymphocytes in vivo. This effect was dose related.


*Br. J. Cancer* (1985), **51**, 55–60

### Recombinant DNA human interferon alpha 2 in advanced breast cancer: a phase 2 trial

N. Padmanabhan[1], F.R. Balkwill[2], J.G. Bodmer[2] & R.D. Rubens[1]

[1]*ICRF Breast Cancer Unit, Guy's Hospital, St Thomas Street, London, SE1 9RT;* [2]*ICRF Laboratories, PO Box No. 123, Lincoln's Inn Fields, London WC2A 3PX, UK.*

**Summary** Effectiveness of recombinant DNA (rDNA) human interferon alpha 2 (IFN alpha 2) in advanced breast cancer was evaluated in 14 patients who had received prior endocrine and/or cytotoxic therapy. After randomization, 7 patients received IFN alpha 2 two million $\mathrm{IU\,m^{-2}\,day^{-1}}$, s.c., 3 times a week (schedule 1) and 7 patients received 50 million $\mathrm{IU\,m^{-2}\,day^{-1}}$, i.v., for 5 consecutive days, every 3 weeks (schedule 2). Treatment duration was 4–21 weeks in schedule 1 and 6–24 weeks (2–8 courses) in schedule 2. Regressions were not achieved with either schedule. Treatment was associated with significant toxicity and was more severe in schedule 2. Dose limiting toxicities were leukopenia, elevation of liver enzymes, hyperglycemia and fatigue.

Serum IFN activity was low or undetectable in patients on schedule 1 and high in patients on schedule 2. At 24 h, serum IFN activity was detectable in only 1/6 patients on schedule 1 as compared to 3/7 patients on schedule 2. IFN neutralizing factors were detected in the serum of only 1 patient prior to treatment but none were detected in any of the patients during or after discontinuation of treatment (4–24 weeks). IFN alpha 2 increased the expression of both HLA class 1 antigens and $\beta 2$ microglobulin in peripheral blood lymphocytes *in vivo*. This effect was dose related.

Response to human interferon alpha (IFN alpha) has been noted in advanced breast cancer in phase 1 trials (Gutterman *et al.*, 1980; Sherwin *et al.*, 1982; Horning *et al.*, 1982). Recombinant DNA (rDNA) human IFN alpha 2 (IFN alpha 2) is a highly purified single subtype in IFN alpha with a specific activity equal to or greater than $10^8\,\mathrm{IU\,mg^{-1}}$ protein. In this paper we report the results of a trial which evaluated the efficacy of IFN alpha 2 in two treatment schedules – a low dose continuous subcutaneous therapy and a high dose pulsed intravenous therapy, in advanced breast cancer. Data obtained on the pharmacokinetics, on the development of serum neutralizing factors to IFN alpha 2 and on the effect of IFN alpha 2 on the level of expression of HLA class 1 antigens and $\beta 2$ microglobulin in peripheral blood lymphocytes are also presented.

### Materials and methods

Female patients with histologically confirmed, evaluable, progressive advanced breast cancer refractory to standard endocrine and/or cytotoxic therapy were considered for the trial. All had a complete clinical examination with measurements and/or photographs of lesions, full blood counts, blood biochemistry, chest radiograph, electrocardiogram and isotopic bone scan with radiographs of regions of increased radionucleide uptake; a liver scan was performed if indicated. Baseline lesions were selected for serial assessment.

Patients were eligible for the trial if the following criteria were fulfilled: Performance status (Karnofsky) $\geq 50\%$; adequate cardiovascular, hepatic [plasma bilirubin $\leq 2$ N (N = upper limit of the normal range) and serum aspartate aminotransferase (AST/SGOT) $\leq 2.5$ N], renal (plasma creatinine $\leq$ N) and bone marrow (hemoglobin $\geq 8\,\mathrm{g\,dl^{-1}}$, white blood cell count $\geq 3\times10^9\,\mathrm{l^{-1}}$ and platelets $\geq 75\times10^9\,\mathrm{l^{-1}}$) function; and no antimitotic therapy or hormone therapy (except corticosteroids at a physiological dose) within past 4 weeks (6 weeks for prior mitomycin C).

Patients were excluded if they had: Non-carcinomatous tumours of the breast, a second malignancy (except localised squamous or basal cell carcinomas of the skin and adequately cone biopsied *in situ* carcinoma of the cervix), central nervous system metastases, serious concomitant non-malignant disease or previous IFN therapy.

The selected patients were then randomized to one of two treatment schedules:

1. IFN alpha 2, $2\times10^6\,\mathrm{IU\,m^{-2}\,day^{-1}}$, s.c., 3 times a week.
2. IFN alpha 2, $50\times10^6\,\mathrm{IU\,m^{-2}\,day^{-1}}$, in 50 ml of saline, given over 30 min, i.v., for 5 consecutive days, every 3 weeks.

Correspondence: N. Padmanabhan
Received 11 July 1984; and in revised form, 26 September 1984.

© The Macmillan Press Ltd., 1985

Treatment was continued until disease progression or unacceptable toxicity was encountered. Responses were assessed by UICC criteria (Hayward et al., 1977).

*Pharmacokinetics*

Blood samples were drawn at 0, 1, 2, 4, 8, 24, 36 and 48 h for patients on schedule 1 and at 0, 1/2, 1, 2, 4, 8, 24 h for patients on schedule 2; during the first week or course of treatment. Serum IFN activity was estimated by the measurement of the inhibition of Semliki forest viral RNA synthesis in human WISH or BT 20 cells. The assay was standardized against the British reference standard for human IFN alpha 69/19 (National Institute of Biological Standards and Controls, London, UK).

*Level of expression of HLA class 1 antigens and β2 microglobulin in peripheral blood lymphocytes*

Blood samples were obtained before and on days 1, 3 and 5 during the first week or course of therapy. The levels of HLA class 1 antigens and β2 microglobulin were assayed using monoclonal anti HLA-ABC and anti β2 microglobulin antibodies.

*IFN neutralizing factors*

Serum samples obtained prior to, at intervals of four weeks during and at the end of treatment were screened for IFN neutralizing factors by the assay of the amount of IFN activity (by a immunoradiometric assay and a bioassay) recovered after the addition of a known amount of IFN alpha 2 to the serum samples. IFN neutralizing factors were considered to be present if the amount of IFN activity recovered was $\leq 50\%$.

### Results

Fourteen patients were entered into the trial, 7 were randomized to schedule 1 and 7 to schedule 2. Table I lists the characteristics, previous treatments and sites of disease for patients on each schedule. All patients had received prior endocrine and/or cytotoxic therapy.

*Toxicity of therapy*

(Tables II and III)

The side effects were more frequent and severe in schedule 2. Patients on schedule 1 developed some degree of tolerance to these side effects while patients on schedule 2 developed little, if any, similar tolerance. Raynaud's phenomenon noted by a patient on schedule 1 during the seventh week of her therapy disappeared after IFN treatment was stopped. The patient on schedule 2 who developed cardiac failure during the third course of treatment had no clinically evident cardiac disease at entry into the trial but had received adriamycin and mitoxantrone (cumulative dose $540\,mg\,m^{-2}$ and $50\,mg\,m^{-2}$ respectively) previously. No arrhythmias or evidence of myocardial infarction were found in serial electrocardiograms but global left ventricular dysfunction with a focal area of akinesis at the apex was found on a gated isotope angiocardiogram suggesting that a cardiomyopathy ± myocardial infarction was the cause of cardiac failure. The patient on schedule 2 who became hyperglycemic was not known to have diabetes before treatment with IFN. A patient on schedule 2 with mild diabetes mellitus and stable blood glucose levels during treatment, developed peripheral neuropathy confirmed by electromyography and nerve conduction studies after receiving 8 courses of IFN alpha 2 in 24 weeks which continued to progress for 6 months after stopping IFN but resolved in the subsequent 6 months. In schedule 1 there were no dose reductions except for a temporary break of 2 weeks in therapy in 1 patient because of fatigue while in schedule 2 dose reduction was necessary in all patients to avoid serious leucopenia (In 26/27

**Table I** Patient characteristics, previous treatments and sites of disease

| | | |
|---|---|---|
| *Patient characteristics* | | |
| *Schedule* | *1* | *2* |
| No. of pts. | 7 | 7 |
| Age at start of IFN median (range) yrs | 55 (39–63) | 62 (50–70) |
| Disease duration median (range) yrs | 5.3 (1.7–22.5) | 4.0 (1.0–18.7) |
| *Previous systemic treatment for breast cancer* | | |
| Chemotherapy only | 1 | 1 |
| Chemotherapy and endocrine therapy | 6 | 6 |
| Endocrine treatments[a] per patient, median (range) | 2 (0–4) | 1 (0–3) |
| Chemotherapy regimens per patient, median (range) | 2(1–3) | 2 (1–3) |
| *Sites of disease* | | |
| Soft tissue only | 3 | 5 |
| Viscera only | 1 | 0 |
| Soft tissues and viscera | 0 | 1 |
| Soft tissue, viscera and bone | 3 | 1 |

[a]Primary, secondary or tertiary endocrine treatment.

**Table II** Toxicity – Incidence

| *Schedule* | *1* *No. of pts* (%) | *2* *No. of pts* (%) |
|---|---|---|
| *General* | | |
| Fever (>37.5°C), chills and rigors, headache, myalgia and backpain | 3–5 (43–71) | 4–7 (57–100) |
| Fatigue | 7 (100) | 7 (100) |
| Somnolence | 2 (29) | 7 (100) |
| Anorexia, nausea and vomiting | 4–5 (57–71) | 6–7 (86–100) |
| Diarrhoea | 1 (14) | 3 (43) |
| *Hematology* | | |
| Depression of hemoglobin ($\leq 10.9\,g\,dl^{-1}$) | 2 (29) | 5 (71) |
| Depression of WBC counts ($\leq 3.9 \times 10^9 l^{-1}$) | 4 (57) | 7 (100) |
| Depression of granulocytes ($\leq 1.9 \times 10^9 l^{-1}$) | 3 (43) | 7 (100) |
| Depression of platelets ($< 100 \times 10^9 l^{-1}$) | 0 | 0 |
| *Liver functions* | | |
| Elevation of serum AST ($\geq 1.26\,N^a$) | 4 (57) | 7 (100) |
| Elevation of serum gamma GT ($\geq 1.26\,N^a$) | 7 (100) | 7 (100) |
| Elevation of alk phos ($\geq 1.26\,N^a$) | 1 (14) | 1 (14) |

*Other*: Schedule 1 – Raynaud's phenomenon 1 pt; Schedule 2 – Cardiac failure 1 pt, hyperglycemia 1 pt and peripheral neuropathy 1 pt.

Toxicity was graded (0–4) according to WHO. criteria (WHO, 1979). This Table shows the number of patients in whom a particular toxicity (grade ≥1) was noted at least once during the course of therapy.
[a]N Upper limit of normal, AST (SGOT) – Aspartate aminotransferase, gamma GT – gamma glutamyl transpeptidase and alk phos – alkaline phosphatase.

courses, median actual cumulative dose expressed as % projected dose of IFN alpha 2 received by patients was 70.3% with a range of 51.7 to 82.3%. The depressed leucocyte counts returned to normal in 3 to 7 days after a course of treatment) and in 2 patients an additional reason for dose reduction was either hyperglycemia or marked elevation serum AST. Treatment had to be stopped, in 2 patients on schedule 1 because of a progressive rise in the levels of serum AST and gamma glutamyl transpeptidase (gamma GT) and in 1 patient on schedule 2 because of cardiac failure. Two patients (1 in each schedule) needed transfusion of red cells for anemia and 2 patients on schedule 2 had an infective episode soon after a course of IFN alpha 2 which responded to antibiotics.

*Response*

All 14 patients were evaluable for response. The treatment duration was 9 (median) and 4–21 (range) weeks for the 7 patients on schedule 1. The disease progressed on treatment in all patients. The 7 patients on schedule 2 received 3 (median) and 2–8 (range) courses of IFN alpha 2 in 6–24 weeks. The disease progressed on treatment in 6 while in 1 patient the disease remained stable after 8 courses (in 24 weeks) of IFN alpha 2.

*Subsequent course of patients*

Nine (5 and 4, schedules 1 and 2 respectively) patients have died of progressive disease after discontinuing treatment with IFN alpha 2. Two out

**Table III** Toxicity – Severity

| *Schedule* | *1* | *2* |
|---|---|---|
| Fever, chills and rigors, headache, myalgia and backpain | 0.4–1.3 | 0.7–2.0 |
| Anorexia, nausea and vomiting | 0.7–1.4 | 1.6–1.9 |
| Fatigue | 2.1 | 1.7 |
| Depression of hemoglobin | 0.4 | 0.9 |
| Depression of white blood cell count | 1.0 | 2.7 |
| Depression of granulocyte count | 0.4 | 2.4 |
| Depression of platelet count | 0 | 0 |
| Elevation of serum AST | 0.6 | 2.4 |
| Elevation of serum gamma GT | 1.7 | 2.1 |
| Elevation of serum alk phos | 0.1 | 0.3 |

Severity of toxicity experienced by patients during the course of treatment expressed on a weighted severity scale. Toxicity graded (0–4) according to the WHO criteria (WHO, 1979), where no such criteria exist grading was from 0 (no symptom) to 4 (very severe toxicity). The worst grade of the side effect experienced by each patient during the course of therapy was taken and the weighted severity was calculated as follows:

Weighted severity = Σ (no. of patients reporting reaction × severity)/no. of patients in the group.

AST (SGOT) – aspartate aminotransferase, gamma GT – gamma glutamyl transpeptidase and alk phos – alkaline phosphatase.

of 7 patients had partial responses to further endocrine treatment (duration of response 5 and 7 mths, 1 pt to prednisolone and 1 pt to aminoglutethimide). Four out of 12 patients had partial responses to further chemotherapy (duration of response median 4.5 mths and range 3 to 6 mths, 1 pt to adriamycin and 3 pts to mitomycin C with vinblastine).

*Pharmacokinetics*

Pharmacokinetic data was obtained in 6 patients on schedule 1 and in 7 patients on schedule 2 during the first week or course of therapy. In patients on schedule 1 the levels of serum IFN activity were either undetectable (1 pt) or low (5 pts). The levels rose gradually to a peak (2.5–25 IU ml$^{-1}$) between 2 and 8 h. At 24 h only one patient had detectable serum IFN activity (6 IU ml$^{-1}$). In patients on schedule 2 the levels of serum IFN activity rose sharply with a peak (280–3904 IU ml$^{-1}$) between 1/2 and 2 h and declined rapidly by 4 h. However, serum IFN activity was still detectable in 3 patients (62–256 IU ml$^{-1}$) at 24 h.

*Effect of IFN alpha 2 on the level of expression of HLA class 1 antigens and β2 microglobulin in peripheral blood lymphocytes*

Data was obtained in 6 patients on schedule 1 and in 7 patients on schedule 2. Only 1 patient on schedule 1 had a twofold increase in the levels of both HLA class 1 antigens and β2 microglobulin. In patients on schedule 2, 6 had a threefold increase in the levels of HLA class 1 antigens and all 7 had three to sixfold increase in the level of β2 microglobulin.

*IFN neutralizing factors*

Only 1 patient out of 14 had detectable IFN neutralizing factors in her serum prior to treatment with IFN alpha 2 but none at 4 weeks (serum IFN activity in this patient during the first course of treatment ranged from 256–1826 IU ml$^{-1}$ with detectable activity of 256 IU ml$^{-1}$ at 34 h). None of the patients had detectable IFN neutralizing factors in the serum samples obtained during and after treatment (median 10 weeks, range 4–24 weeks).

### Discussion

Treatment with IFN alpha 2 was associated with significant toxicity which was dose related. Depression of bone marrow and hepatic dysfunction were the major dose limiting factors. Central nervous system toxicity was limited to somnolence and the serious toxicity noted with the use of much higher doses of IFN was not seen (Rohatiner *et al.*, 1983; Nethersell *et al.*, 1984). Hyperglycemia (data on file with Schering-Plough corp.), Raynaud's phenomenon (Sangster *et al.*, 1983), cardiac toxicity (Oldham, 1983), peripheral neuropathy (Gutterman *et al.*, 1982) and other toxicities (Gutterman *et al.*, 1980, 1982; Sherwin *et al.*, 1982, 1983; Horning *et al.*, 1982; Borden *et al.*, 1982; Edelstein *et al.*, 1983) noted in this trial have been reported previously.

The lack of antitumour activity for IFN alpha 2 as noted in this trial differs from the results of studies *in vitro*, in animal tumour model systems and from the results of other clinical trials (Gutterman *et al.*, 1980; Sherwin *et al.*, 1982; Horning *et al.*, 1982; Borden *et al.*, 1982). Two possible explanations can be given to account for this discrepancy. First, it is possible that a mixture of species of IFNs alpha is more active than a single species, as the phase 2 trial (Borden *et al.*, 1982) which evaluated leukocyte derived IFN (a mixture of species of IFNs alpha) in advanced breast cancer found 5 partial responses in 23 patients (median duration of response 59 days, range 14–176 days) while the trials which evaluated a single pure species of IFN alpha found none (current trial and Sherwin *et al.*, 1983 – evaluated rDNA human IFN alpha A which is essentially identical but differs by one amino acid from rDNA IFN alpha 2, see Pestka, 1983).

Second, it is possible that the previous cytotoxic therapy received by patients in this trial had a role in determining the resistance of their tumours to IFN. This explanation is supported by the fact that all the patients in this trial and in the trial reported by Sherwin *et al.* (1983), had received prior cytotoxic ± endocrine therapy whereas patients included in the trial reported by Borden *et al.* (1982), had either received no prior systemic therapy or were responders to prior endocrine therapy only and in the trial reported by Nethersell *et al.* (1984, evaluated rDNA human IFN alpha A) the only 2 responders (partial responses, noted at 4 wks not maintained at 12 wks) were patients who had not received prior cytotoxic therapy but were responders to prior endocrine therapy. The observations that: (a) enough serum IFN activity was present in all patients to produce some degree of toxicity, (b) serum IFN activity was detectable in 12 patients out of the 14, (c) 8 patients had changes in the level of expression of HLA class 1 antigens and/or $\beta 2$ microglobulin and (d) IFN neutralizing factors were noted in only 1 patient; suggest that tumour resistance to IFN alpha 2 is the probable explanation for the lack of response noted in this trial. The observation that useful responses were obtained to further chemo and endocrine therapy suggests that IFN alpha 2 did not have an adverse influence on the outcome of subsequent treatment.

The increase in the level of expression of HLA class 1 antigens and $\beta 2$ microglobulin in peripheral blood lymphocytes induced by IFN alpha 2 confirms that these changes seen *in vitro* (Hokland *et al.*, 1981), occur *in vivo*. It may be possible to define an optimum dose for IFN using this system as this effect seems to be dose related.

The lack of response to IFN alpha 2 in the 14 patients evaluated in this trial suggests that the rate of response to this drug when used as a single agent in advanced breast cancer in either dose schedule, is unlikely to be greater than 35% in patients who have received prior cytotoxic ± endocrine therapy. If these results are considered in conjunction with the results obtained (in patients who had received prior cytotoxic ± endocrine therapy) in other phase 2 trials (Sherwin *et al.*, 1983; Nethersell *et al.*, 1984) the response rate for IFN alpha 2 in this defined set of patients is unlikely to be greater than 20%. As some studies suggest synergism between IFN and cytotoxic drugs (Balkwill & Moodie, 1984), the future role of this drug in advanced breast cancer will have to be defined by trials to evaluate such combinations.

We thank Miss H.A. Band, Mr T.H. Duhig and Mrs D.B. Griffin, IFN Laboratory, I.C.R.F., Lincoln's Inn Fields, for their help with the IFN assays; Miss Y. Reid, Tissue Antigen Laboratory, I.C.R.F., Lincoln's Inn Fields, for her help with the studies on the expression of HLA antigens and $\beta 2$ microglobulin and Schering-Plough Corporation for the rDNA human IFN alpha 2 and the studies on serum IFN neutralizing factors. We are grateful to Mr J.L. Hayward, I.C.R.F. Breast Cancer Unit, for his comments on the manuscript.

**References**

BALKWILL, F.R. & MOODIE, E.M. (1984). Positive interactions between human interferon and cyclophosphamide or adriamycin in human tumour model system. *Cancer Res.*, **44**, 904.

BORDEN, E.C., HOLLAND, J.F., DAO, T.L. & 4 others. (1982). Leukocyte derived interferon alpha in human breast carcinoma. *Ann. Intern. Med.*, **97**, 1.

EDELSTEIN, M.B., SCHELLEKENS, H., LAURENT, T. & GAUCI, L. (1983). A phase 1 clinical tolerance study of rDNA alpha 2 human interferon in patients with non-reticuloendothelial system malignancies. *Eur. J. Cancer Clin. Oncol.*, **19**, 891.

GUTTERMAN, J.U., BLUMENSCHEIN, G.R., ALEXANIAN, R. & 9 others. (1980). Leukocyte interferon induced tumor regression in human metastatic breast cancer, multiple myeloma and malignant lymphoma. *Ann. Intern. Med.*, **93**, 399.

GUTTERMAN, J.U., FINE, S., QUESADA, J. & 10 others. (1982). Recombinant leukocyte A interferon: Pharmacokinetics, single dose tolerance and biologic effects in cancer patients. *Ann. Intern. Med.*, **96**, 549.

HAYWARD, J.L., CARBONE, P.P., HEUSON, J.C., KUMAOKA, S., SEGALOFF, A. & RUBENS, R.D. (1977). Assessment of response to therapy in advanced breast cancer. *Eur. J. Cancer*, **13**, 89.

HOKLAND, M., HERON, I. BERG, K. (1981). Increased expression of $\beta 2$ microglobulin and histocompatibility antigens on human lymphoid cells induced by interferon. *J. IFN. Res.*, **1**, 483.

HORNING, S.J., LEVINE, J.F., MILLER, R.A., ROSENBERG, S.A. & MERIGAN, T.C. (1982). Clinical and immunologic effects of recombinant leukocyte interferon in eight patients with advanced cancer. *J.A.M.A.*, **247**, 1718.

NETHERSELL, A., SMEDLEY, H., KATRAK, M., WHEELER, T. & SIKORA, K. (1984). Recombinant interferon in advanced breast cancer. *Br. J. Cancer*, **49**, 615.

OLDHAM, R.R. (1983). Toxic effects of interferon (letter). *Science*, **219**, 902.

PESTKA, S. (1983). The purification and manufacture of human interferons. *Sci. Am.*, **249**, 28.

ROHATINER, A.Z.S., PRIOR, P.F., BURTON, A.C., SMITH, A.T., BALKWILL, F.R. & LISTER, T.A. (1983). Central nervous system toxicity of interferon. *Br. J. Cancer,* **47,** 419.

SANGSTER, G., KAYE, S.B., CALMAN, K.C. & TOY, J.L. (1983). Cutaneous vasculitis associated with interferon (letter). *Eur. J. Cancer Clin. Oncol.,* **19,** 1647.

SHERWIN, S.A., KNOST, J.A., FEIN, S. & 6 others. (1982). A multiple dose phase 1 trial of recombinant leukocyte A interferon in cancer patients. *J.A.M.A.,* **248,** 2461.

SHERWIN, S.A., MAYER, D., OCHS, J.J. & 5 others. (1983). Recombinant leukocyte A interferon in advanced breast cancer – Results of a phase 2 efficacy trial. *Ann. Intern. Med.,* **98,** (part 1), 598.

W.H.O. (1979). W.H.O. handbook for reporting results of cancer treatment, *W.H.O. Offset Publication No. 48,* W.H.O., Geneva, 1979.